# Supplementary material for: Prevalence and predictors of magnesium imbalance among critically ill diarrheal children and their outcome in a developing country
Source: PLoS One. 2023 Dec 15;18(12):e0295824. doi: 10.1371/journal.pone.0295824 (PMC10723721; doi:10.1371/journal.pone.0295824)
Supplement: S1 Table — (DOCX) [file pone.0295824.s002.docx]

**Supplementary Table 1**. Electrolytes distribution other than magnesium of normomagnesemia and hypomagnesemia participants

| **Characteristics** | **Normomagnesemia (n=344)** | **Hypomagnesemia (n=29)** | **mOR (95% CI)** | **P value** |
| --- | --- | --- | --- | --- |
| **Sodium** | 134.8 ± 11.9 | 132.3 ± 7.5 | 0.98 (0.95-1.02) | 0.323 |
| **Potassium** | 4.00 ± 1.12 | 3.9 ± 0.91 | 0.92 (0.67-1.26) | 0.608 |
| **Chloride** | 107.6 ± 13.7 | 103.3 ± 7.7 | 0.98 (0.95-1.01) | 0.135 |
| **HCO_3_^-^** | 14.94 ± 5.19 | 16.92 ± 3.26 | 1.07 (0.997-1.14) | 0.062 |
| **Calcium** | 2.17 ± 0.28 | 1.87 ± 0.28 | 0.04 (0.01-0.14) | <0.001 |

**Supplementary Table 2**. Electrolytes distribution other than magnesium of normomagnesemia and hypermagnesemia participants

| **Characteristics** | **Normomagnesemia (n=344)** | **Hypermagnesemia (n=184)** | **mOR (95% CI)** | **P value** |
| --- | --- | --- | --- | --- |
| **Sodium** | 134.8 ± 11.9 | 149.8 ± 24.4 | 1.05 (1.04-1.06) | <0.001 |
| **Potassium** | 4.00 ± 1.12 | 4.14 ± 1.54 | 1.09 (0.95-1.26) | 0.222 |
| **Chloride** | 107.6 ± 13.7 | 125.7 ± 26.0 | 1.05 (1.04-1.06) | <0.001 |
| **HCO_3_^-^** | 14.94 ± 5.19 | 10.98 ± 5.89 | 0.87 (0.84-0.90) | <0.001 |
| **Calcium** | 2.17 ± 0.28 | 2.22 ± 0.29 | 2.16 (1.12-4.16) | 0.022 |

**Supplementary Table 3**. Comparison of disease course of hypomagnesemia with normomagnesemia children during the hospital stay

| **Characteristics** | **Normomagnesemia (n=344)** | **Hypomagnesemia (n=29)** | **mOR (95% CI)** | **P value** |
| --- | --- | --- | --- | --- |
| **Ventilator support required** | 24 (7.0) | 4 (13.8) | 2.1 (0.69-6.63) | 0.190 |
| **Duration of ICU stay (median, IQR)** | 2 (1, 3) | 2 (1, 3) | 1.01 (0.92-1.12) | 0.793 |
| **Outcome-** Discharge | 266 (77.3) | 18 (62.1) | Reference | |
| LAMA^1^ or referred | 53 (15.4) | 7 (24.1) | 1.95 (0.78-4.90) | 0.155 |
| Death | 25 (7.3) | 4 (13.8) | 2.36 (0.74-7.53) | 0.145 |

^1^ LAMA- Left against medical advice

**Supplementary Table 4**. Comparison of disease course of hypermagnesemia with normomagnesemia children during the hospital stay

| **Characteristics** | **Normomagnesemia (n=344)** | **Hypermagnesemia (n=184)** | **mOR (95% CI)** | **P value** |
| --- | --- | --- | --- | --- |
| **Ventilator support required** | 24 (7.0) | 23 (12.5) | 1.90 (1.04-3.48) | 0.036 |
| **Duration of ICU stay (median, IQR)** | 2 (1, 3) | 3 (1, 4) | 1.02 (0.97-1.07) | 0.422 |
| **Outcome-** Discharge | 266 (77.3) | 106 (57.6) | Reference | |
| LAMA^1^ or referred | 53 (15.4) | 55 (29.9) | 2.60 (1.68-4.04) | <0.001 |
| Death | 25 (7.3) | 23 (12.5) | 2.31 (1.26-4.25) | 0.007 |

^1^ LAMA- Left against medical advice
